# Multidimensional prognostic index and the risk of fractures: an 8-year longitudinal cohort study in the Osteoarthritis Initiative

**DOI:** 10.1007/s11657-021-01015-3

**Published:** 2021-12-14

**Authors:** Nicola Veronese, Lee Smith, Ekaterini Zigoura, Mario Barbagallo, Ligia J. Dominguez, Antonella Barone, Alberto Cella, Cyrus Cooper, Renè Rizzoli, Jean-Yves Reginster, Stefania Maggi, Alberto Pilotto

**Affiliations:** 1 Geriatric Unit, Department of Internal Medicine and Geriatrics, University of Palermo, Via del Vespro, 141 90127 Palermo, Italy; 2 Biochemistry Department, College of Science, King Saud University, Riyadh, Saudi Arabia; 3 The Cambridge Centre for Sport and Exercise Sciences, Anglia Ruskin University, Cambridge, UK; 4 Department Geriatric Care, Orthogeriatrics and Rehabilitation, Frailty Area, E.O. Galliera Hospital, Genova, Italy; 5 MRC Lifecourse Epidemiology Unit, Southampton General Hospital, University of Southampton, Southampton, UK; 6 NIHR Musculoskeletal Biomedical Research Unit, University of Oxford, Oxford, UK; 7 Division of Bone Diseases, Faculty of Medicine, Geneva University Hospitals, Geneva, Switzerland; 8 WHO Collaborating Centre for Public Health Aspects of Musculoskeletal Health and Aging, Liège, Belgium; 9 Department of Public Health, Epidemiology and Health Economics, University of Liège, CHU Sart Tilman B23, 4000 Liège, Belgium; 10 Biochemistry Department, College of Science, King Saud University, Riyadh, Kingdom of Saudi Arabia; 11 Aging Branch, Neuroscience Institute, National Research Council, Padua, Italy; 12 Department of Interdisciplinary Medicine, University of Bari, Bari, Italy

**Keywords:** Multidimensional prognostic index, Comprehensive geriatric assessment, Fractures, Osteoarthritis Initiative

## Abstract

***Summary*:**

In this longitudinal study, with a follow-up of 8 years, multidimensional prognostic index (MPI), a product of the comprehensive geriatric assessment, significantly predicted the onset of fractures in older people affected by knee osteoarthritis.

**Purpose:**

Frailty may be associated with higher fracture risk, but limited research has been carried out using a multidimensional approach to frailty assessment and diagnosis. The present research aimed to investigate whether the MPI, based on comprehensive geriatric assessment (CGA), is associated with the risk of fractures in the Osteoarthritis Initiative (OAI) study.

**Methods:**

Community-dwellers affected by knee OA or at high risk for this condition were followed-up for 8 years. A standardized CGA including information on functional, nutritional, mood, comorbidity, medication, quality of life, and co-habitation status was used to calculate the MPI. Fractures were diagnosed using self-reported information. Cox’s regression analysis was carried out and results are reported as hazard ratios (HRs), with their 95% confidence intervals (CIs), adjusted for potential confounders.

**Results:**

The sample consisted of 4024 individuals (mean age 61.0 years, females = 59.0%). People with incident fractures had a significant higher MPI baseline value than those without (0.42 ± 0.18 vs. 0.40 ± 0.17). After adjusting for several potential confounders, people with an MPI over 0.66 (HR = 1.49; 95%CI: 1.11–2.00) experienced a higher risk of fractures. An increase in 0.10 point in MPI score corresponded to an increase in fracture risk of 4% (HR = 1.04; 95%CI: 1.008–1.07). Higher MPI values were also associated with a higher risk of non-vertebral clinical fractures.

**Conclusion:**

Higher MPI values at baseline were associated with an increased risk of fractures, reinforcing the importance of CGA in predicting fractures in older people affected by knee OA.

## Introduction

Frailty is a common condition in older people, affecting approximately one in ten people after 60 years of age [1]. Frailty is associated with several negative outcomes including disability [2], higher risk of hospitalization [3], and mortality [4], including mortality for the recent COVID-19 epidemic [5]. Recent literature has also reported that frailty might be considered a potential risk factor for other medical conditions including cardiovascular disease [6], osteoarthritis [7], and osteoporotic fractures, [8] a leading cause for disability and mortality in older people [9]. In a systematic review and meta-analysis of six studies involving 96,564 older community-dwelling people, frailty was significantly associated with future risk of fractures [8]. Even if frailty is a well-recognized entity in geriatric medicine, several definitions are available, mainly based on physical performance criteria [10]. For example, using the definition proposed in the Study of Osteoporotic Fractures (i.e., significant weight loss, inability to rise from a chair 5 times without using arms, and reduced energy level), frailty predicted the onset of fractures [11], similarly to the criteria more widely used in geriatric medicine and proposed by Fried et al. in the Cardiovascular Health Study (CHS) [12].

However, no study to date has explored the importance of comprehensive geriatric assessment (CGA) and the multidimensional model in predicting fracture risk [13]. In this context, the multidimensional prognostic index (MPI) [14] is a well-calibrated tool with a relevant discrimination and accuracy for mortality in hospital [15] and in primary care settings [16]. Among all indexes used in geriatrics for clinical decision-making, the MPI is the only tool that allows for the exploration of multiple domains, relating to general health, functional, cognitive, and nutritional status, as well as social aspects, using standardized and extensively validated rating scales [17]. Furthermore, MPI is significantly correlated with several negative outcomes in older people, in particular mortality and (re)hospitalization [14, 15, 18, 19]. Recent literature has reported a significant association between MPI and other conditions, such as poor quality of life [20], cardiovascular disease [21], and depression [22].

However, to the best of our knowledge, no study has explored whether higher MPI values are associated with a higher risk of fractures. Given this background, the present study aimed to investigate the association between MPI scores and incident fractures in a large cohort of North American adults followed up over 8 years, participating to the Osteoarthritis Initiative (OAI).

## Materials and methods

### Data source and subjects

Data from the Osteoarthritis Initiative (OAI) database were used. The participants were included across four clinical sites in the USA (Baltimore, MD; Pittsburgh, PA; Pawtucket, RI; and Columbus, OH) between February 2004 and May 2006. Inclusion criteria were as follows: (1) had knee osteoarthritis (OA) with knee pain for a 30-day period in the past 12 months including all grades of severity or (2) were at high risk of developing knee OA (e.g., overweight/obese [body mass index, BMI ≥ 25 kg/m^2^], family history of knee OA) [23]. For the aims of this research, the data were collected at baseline and during the following evaluations over 8 years of follow-up. The presence of fractures in the OAI was recorded, other than the first evaluation, after 1, 2, 3, 4, 6, and 8 years from baseline. All participants provided written informed consent. The OAI study was given full ethics approval by the institutional review board of the OAI Coordinating Center, University of California in San Francisco.

### Calculation of the MPI

Originally, the MPI was built according to eight different scales, i.e., disability in basic and instrumental activities of daily living, using the Katz [24] and Lawton-Brady [25] indexes, respectively, nutritional domain, investigated with the mini-nutritional assessment [26], severity of comorbidities [27], number of drugs taken daily, risk of pressure sores [28], cognitive performance [29], and social aspects. In the OAI, since some of these scales were not available, the MPI was calculated as reported in other studies using the same database [21, 30]. Six domains were assessed by using standardized CGA scales: (1) physical functioning, through the Western Ontario and McMaster Universities Osteoarthritis (WOMAC) Index [31]; (2) physical activity, measured through the Physical Activity Scale for the Elderly scale (PASE) [32]; (3) nutritional aspects, evaluated using body mass index (BMI); (4) comorbidity, assessed by the Charlson Comorbidity Index score [24]; (5) the number of medications used; (6) co-habitation status was reported, categorized as living alone (yes vs. no); (7) the presence of depressive symptoms, using the Center for Epidemiologic Studies-Depression (CES-D) Scale [33]; and (8) quality of life assessed through a specific subscale of the Knee injury Osteoarthritis Outcome Score (KOOS) [34].

This modified MPI, obtained as weighted sum of each domain, ranged from 0.0 (low risk) to 1.0 (highest risk). MPI was categorized into three statistically different risk groups of fracture risk (low risk 0–0.33, moderate risk 0.34–0.66, and severe risk > 0.66), similar to the original division of this score.

The changes of the MPI during follow-up were evaluated at V03, V06, V08, and V10 since information regarding comorbidity were available only at these evaluations.

### Outcome: fracture assessment

The presence of fractures at baseline and during follow-up was ascertained through self-reported history of fractures [35]. The primary outcome was considered the incidence of any fracture.

### Covariates

Other than age and sex, we identified several potential confounders in the possible relationship between MPI and incident fractures. These included (1) smoking habits, categorized as “previous/current” vs. never; (2) ethnicity, categorized as whites vs. others; (3) educational level, categorized as “degree” vs. others; (4) yearly income, divided as < vs. ≥ $50,000 or missing data; (5) use of anti-osteoporotic medications at baseline (hormones [including raloxifene], bisphosphonates, teriparatide); (6) number of alcoholic drinks in a typical week; (7) the presence of any fracture at the baseline evaluation; (8) the use of some medications that seem to be associated with a higher risk of fractures, such as SSRI (selective serotonin reuptake inhibitors) [36] and pump inhibitors [37]; (9) vitamin D intake, calculating the sum between that introduced with the diet and with the supplementations; (10) the presence of knee pain evaluated using the highest value of the two WOMAC pain subscale indexes, assessed in both knees [31]; and (11) the job strain, classified as worker or unemployed/retired.

### Statistical analyses

Data on continuous variables were normally distributed according to the Kolmogorov–Smirnov test. Data were presented as means and standard deviation values (SD) for quantitative measures (if normally distributed) and absolute numbers (and percentages) for the discrete variables, by MPI categories (≤ 0.33; 0.34–0.66; > 0.66). Levene’s test was used to test the homoscedasticity of variances and, if its assumption was violated, Welch’s ANOVA was used. *p* values for trends were calculated using the Jonckheere-Terpstra test for continuous variables and the Mantel–Haenszel chi-square test for categorical ones.

Cox’s regression analysis was run, taking the MPI at the baseline (in categories or as increase in 0.10 points) as the exposure variable and incident fractures as the outcome variable. For missing data regarding the changes in MPI during the follow-up period, we used a multiple imputation approach. The data were reported as hazard ratios (HRs) with their 95% confidence intervals (CIs), adjusted for the confounders mentioned before. These confounders were chosen since they were associated, using a Cox regression analysis, with incident fractures, using a *p* value < 0.20 as criterion for entering in the model. Since a large number of covariates were included in our analyses and for avoiding the risk of collinearity, a backward logistic regression model was applied to better select the factors more predictive of incident fractures in our cohort.

Moreover, to test the robustness of our results, we ran several sensitivity analyses (i.e., median age, gender, use of anti-osteoporotic medications, presence of fractures already at the baseline), but the *p* for interaction for MPI by these factors in predicting incident fractures was > 0.05 for all these strata. Since self-reporting of vertebral fractures might not provide an accurate picture of the actual number of fractures, we conducted a further sensitivity analysis, looking only at non-vertebral fractures (i.e., forearm and hip).

All the analyses were performed using the SPSS 21.0 for Windows (SPSS Inc., Chicago, IL). All statistical tests were two-tailed and statistical significance was assumed for a *p* value < 0.05.

## Results

### Sample selection

The OAI dataset included, at baseline evaluation, a total of 4796 individuals. After removing 346 for which data regarding MPI at baseline were not available and 426 lost at follow-up (i.e., information regarding incident fractures were not recorded), 4024 participants were finally included.

### Baseline characteristics

The 4024 participants included were more women (59.0%), with a mean age of 61.0 years (± 9.1 years; range: 45–79 years). The mean MPI at baseline was 0.40 ± 0.17 (range: 0.0–1.0).

Table 1 illustrates the characteristics by baseline MPI values. Participants in the higher MPI category (MPI-3, MPI 0.67–1.00) (*n* = 255) were significantly more likely to be female, older, non-white, a smoker, less educated, and less wealthy than those in the lowest category of MPI (< 0.33) (*n* = 1451). People in the MPI-3 group used more frequently PPIs or SSRIs, and reported significant higher values of WOMAC pain than their counterparts with lower values.

**Table 1:** Descriptive statistics of participants’ characteristics according to their baseline MPI value

		MPI-1 (0.00–0.33) (*n* = 1451)	MPI-2 (0.34–0.66) (*n* = 2318)	MPI-3 (0.67–1.00) (*n* = 255)	*p* values for trend
Sex, *n* (%)	F	738 (50.9)	1448 (62.5)	189 (74.1)	< 0.0001
	M	713 (49.1)	870 (37.5)	66 (25.9)	
Age, mean (SD)		60.0 (9.1)	61.1 (9.1)	61.5 (8.9)	< 0.0001
Number of alcoholic drinks per week, mean (SD)		1.9 (1.5)	1.7 (1.5)	1.3 (1.4)	< 0.0001
Yearly income > $50,000/year, *n* (%)		1086 (74.8)	1289 (55.6)	69 (27.1)	< 0.0001
Whites, *n* (%)		1284 (88.5)	1827 (78.8)	157 (61.6)	< 0.0001
College or higher education, *n* (%)		550 (37.9)	662 (28.6)	38 (14.9)	< 0.0001
Smoking status, *n* (%)		593 (40.9)	1123 (48.4)	140 (54.9)	< 0.0001
Current employer, *n* (%)		1037 (71.5)	1381 (59.6)	107 (42.0)	< 0.0001
Use of SSRI, *n* (%)		71 (4.9)	219 (9.5)	65 (25.2)	< 0.0001
Use of PPI, *n* (%)		129 (8.9)	326 (14.1)	58 (22.8)	< 0.0001
Intake of vitamin D (foods and supplementations)		415 (247)	409 (250)	384 (255)	0.17
WOMAC pain subscale		1.86 (2.64)	3.84 (2.58)	7.07 (4.27)	< 0.0001
Use of anti-osteoporotic medications, *n* (%)		282 (19.4)	579 (25.0)	74 (29.0)	< 0.0001
Presence of osteoporotic fractures, *n* (%)		236 (16.3)	417 (18.1)	61 (23.9)	< 0.0001
Living alone, *n* (%)		1313 (90.5)	1723 (74.3)	99 (38.8)	< 0.0001
CES-D, mean (SD)		2.5 (3.0)	1.7 (6.5)	17.5 (9.3)	< 0.0001
PASE, mean (SD)		197 (86)	147 (74)	106 (53)	< 0.0001
Comorbidity, mean (SD)		0.11 (0.37)	0.32 (0.72)	1.10 (1.33)	< 0.0001
Number of drugs, mean (SD)		1.9 (1.9)	3.1 (2.6)	5.4 (3.7)	< 0.0001
KOOS – QoL, mean (SD)		77 (19)	63 (21)	47 (21)	< 0.0001
BMI, mean (SD)		26.9 (3.9)	29.2 (3.9)	31.8 (5.4)	< 0.0001

*p* values for trends were calculated using the Jonckheere-Terpstra test for continuous variables and the Mantel–Haenszel chi-square test for categorical ones

*BMI*, body mass index; *CES-D*, Center for Epidemiologic Studies-Depression; *KOOS*, Knee Injury and Osteoarthritis Outcome Score; *MPI*, multidimensional prognostic index; *PASE*, Physical Activity Scale for Elderly; *QoL*, quality of life; *SD*, standard deviation; *WOMAC*, Western Ontario and McMaster University; *SSRI*, selective serotonin reuptake inhibitors; *PPI*, pump inhibitors

### Follow-up analyses

During the 8 years of follow-up, 723 participants (= 18.0%) experience a fracture. Among the fractures, for 479 the site was not specified, 164 were reported at the forearm or hip, and 70 were vertebral. Among the 723 people with incident fracture, 244 (16.8%) were in the MPI-1 group, 411 (17.7%) in the MPI-2 group, and 68 (26.7%) in the MPI-3 group. People with incident fractures had a significant higher MPI baseline value than those without this condition (0.42 ± 0.18 vs. 0.40 ± 0.17, *p* = 0.005). Overall, 210 participants (29.4%) with an incident fracture had a diagnosis of fracture already at the baseline. Of them, 57 (27.1%) were classified in the MPI-1, 130 (61.9%) in the MPI-2, and 23 (11.0%) in the MPI-3 group.

**Fig. 1:**
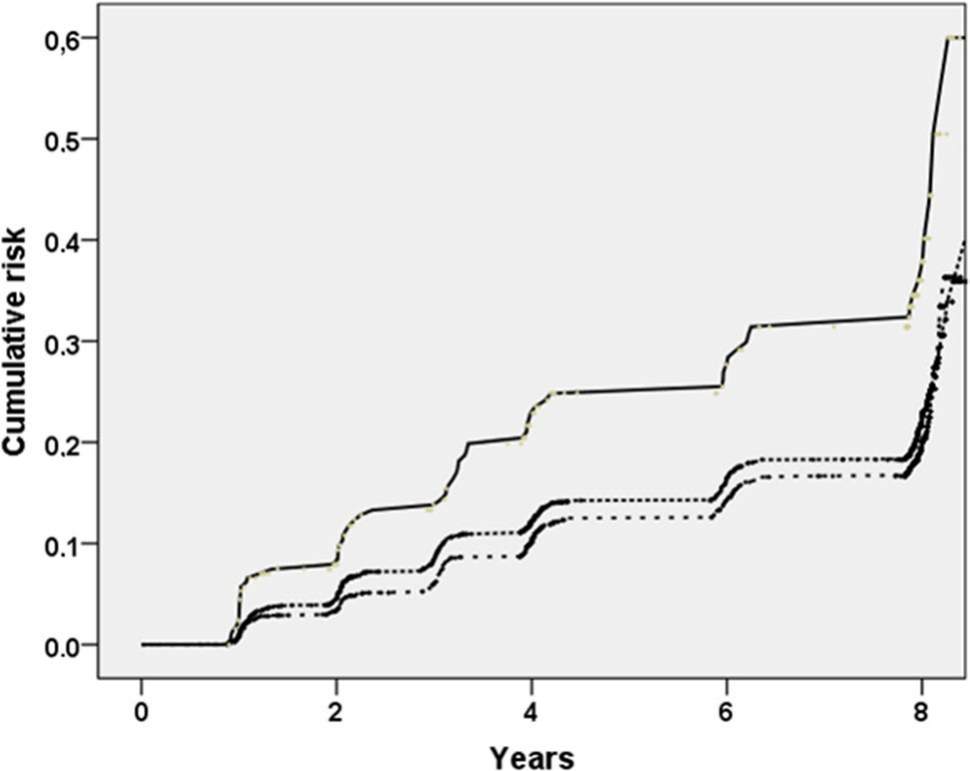
Cumulative incidence of fractures during the 8 years of follow-up, by MPI categories*.* The MPI-3 group is represented by the continuous line (upper line), MPI-2 and MPI-1 by dashed lines

Table 2 shows the Cox regression analysis taking MPI as exposure and incident fractures during the 8 years of follow-up as outcome. People in the MPI-3 group had a doubled incidence of fractures than those in the MPI-1 group (25 in MPI-1 and 49 in MPI-3 events per 1000-year) (Fig. 1). After adjusting for several potential confounders at the baseline evaluation, people in the MPI-3 group (HR = 1.49; 95%CI: 1.11–2.00; *p* = 0.008) had a higher risk of incident fractures (Table 2).

**Table 2:** Association between MPI and incident fractures during 8 years of follow-up

		Fractures (incidence rate, per 1000-year)	Unadjusted HR (95%CI)	HR^1^ (95%CI)
MPI (× 0.10 increase)		-	1.08 (1.03–1.13) (*p* = 0.001)	1.04 (1.008–1.07) (*p* = 0.03)
MPI	MPI-1	25	1	1
	(0.00–0.33)	(22–29)	[reference]	[reference]
	MPI-2	28	1.11 (0.94–1.30)	1.01 (0.86–1.19)
	(0.34–0.66)	(25–31)	(*p* = 0.21)	(*p* = 0.88)
	MPI-3	49	1.85 (1.41–2.43)	1.49 (1.11–2.00)
	(0.67–1.00)	(38–62)	(*p* < 0.0001)	(*p* = 0.008)

^1^Hazard ratios are adjusted for age; sex; ethnicity; education; smoking status; monthly income; use of anti-osteoporotic medications (hormones, bisphosphonates, teriparatide); number of alcoholic drinks in a typical week; the presence of any fracture at the baseline evaluation; the use of SSRI (selective serotonin reuptake inhibitors) and/or pump inhibitors; vitamin D intake (diet and the supplementations); the presence of knee pain; and the job strain, classified as worker vs. unemployed/retired

*MPI*, multidimensional prognostic index; *HR*, hazard ratio; *CI*, confidence intervals

In a sensitivity analysis, after removing 70 incident vertebral fractures and 479 for which the site was not specified, 164 forearm and hip fractures were recorded: 54 participants had a prevalent fracture at baseline, while 55, 93, and 16 were classified in MPI-1, 2, and 3, respectively. Compared to people in MPI-1, people in MPI-3 experienced a higher risk of fracture (HR = 1.69; 95%CI: 1.04–3.03; *p* = 0.03).

## Discussion

During the 8 years of follow-up, we found that MPI at baseline may predict the onset of fractures in community-dwelling participants affected by knee OA or at high risk for this condition. The incidence of fractures was nearly doubled in people in the MPI-3 group compared to that in the MPI-1 group and these findings remain significant after adjusting our analyses for several potential confounders. The subjects with incident fractures had a significantly higher mean MPI value of 0.02 points. While this difference is small, a previous study in hospitalized older people reported that a similar difference in MPI between admission and discharge was able to predict mortality [38].

People having higher MPI values at baseline had a significantly higher presence of common risk factors for fractures than people with lower values of MPI, such as female gender, lower educational level, and higher presence of smoking and prevalent fractures. However, also after adjusting for these and other potential confounders, the association between MPI and incident fractures remains significant. There are several explanations that may justify the present findings. First, it has been reported that frail people might have several cellular (such as deoxyribonucleic acid damage and shorter telomere length) [39] and bio-humoral alterations (e.g., higher oxidative stress and inflammatory levels) [40] that can increase fracture risk [41]. People with higher MPI values can have a higher risk of falls [30], an independent risk factor for fractures [42].

Other investigations have already reported the importance of frailty in predicting fractures. A seminal paper, published more than 20 years ago, found that SOF and CHS indexes have a similar accuracy in predicting fractures, indicating the importance of detecting frailty in older people [11]. However, CGA may add other important information in older people, such as social aspects and medication history, not considered in the two indexes mentioned before. For example, in one study of 2033 patients followed-up for 1 year and comparing tools commonly used in geriatric medicine for frailty identification, the MPI derived from the CGA had the highest accuracy in predicting mortality, in hospitalized older people [43]. We believe that our findings are of importance as they suggest that CGA is an essential step in osteoporosis management. From years, for example, CGA is integrated with orthopedics in hip fracture management [44]. However, our study further indicates that CGA can be useful in the prevention of future fractures’ risk since it identifies people at higher risk of fractures. Our findings further strengthen the concept that frailty is significantly associated with fractures, as already shown in other works diagnosing frailty using physical performance criteria [8].

We can discuss the suitability of MPI in predicting fractures, compared to the most common tools used in this field for predicting these outcomes. For example, the FRAX score is a common tool used in the management of osteoporosis: this tool determines fracture probability in individuals by integrating important individual clinical risk factors, giving more information than BMD alone [45]. Even if it is not possible in the OAI study to compare the accuracy of the FRAX score vs. the MPI in predicting frailty, the first one is specific for the prediction of fractures, while MPI can be used for predicting mortality and other medical conditions. For example, in the OAI study, we have already reported that the same MPI used for the aims of this work is able to predict the onset of cardiovascular disease and falls [21, 30]. Future research should assess if MPI is able to increase the accuracy in predicting fractures of the FRAX score.

The findings from this study should be interpreted in light of its limitations. First, the OAI study includes only people who already have or are at high risk of knee OA, being overweight or obese. Thus, whether our results can be applied to the general population should be verified in general population studies. Second, fractures were self-reported by the patients and not validated by specialists or medical records. This may lead to an under-representation of fractures [46] that are often asymptomatic. Some studies showed that for clinical fractures the accuracy of self-reported fractures is accurate and similar to radiological records, but probably there is an underestimation of some non-clinical fractures, especially vertebral ones [47, 48]. Furthermore, no data about bone mineral density (BMD) is available and this could introduce another confounding factor into our findings, even if it is difficult to say in which direction. It is widely known, in fact, that BMD is among the most important predictors of bone fractures and it is included in several prognostic scores for predicting fractures [49]. Fourth, since it is a retrospective study, the construction of the MPI was revised using the available data in the dataset and not using the standard definition of this tool. However, how this choice can impact the generalizability of the data is hard to determine.

In conclusion, our data suggest that higher MPI values at baseline might be associated with an increased risk of fractures over 8 years of follow-up, further suggesting the importance of CGA in predicting fractures in older people having knee osteoarthritis. Other longitudinal studies, in general population, are needed to confirm our findings.

## Data Availability

The data of the OAI are freely available at https://www.niams.nih.gov/grants-funding/funded-research/osteoarthritis-initiative.

## Code availability

All analyses were performed using the SPSS 20.0 for Windows (SPSS Inc., Chicago, IL).
